# Metabolic, Gastric, and Psychosocial Correlates of Carbohydrate-Containing Clear-Fluid Intake in Children Undergoing Adenotonsillectomy: A Prospective Cohort Study

**DOI:** 10.3390/nu18213684

**Published:** 2026-11-06

**Authors:** Gamze Atas, Hakan Gokalp Tas, Talha Karatas, Ilker Atas, Tulay Ceren Olmezturk Karakurt, Taha Emre Otugen, Ufuk Kuyrukluyildiz

**Affiliations:** 1 Department of Anesthesiology and Reanimation, Mengucek Gazi Training and Research Hospital, Erzincan 24000, Turkey; dr.gamzeatas@gmail.com (G.A.); tahaemreotugen@gmail.com (T.E.O.); 2 Department of Anesthesiology and Reanimation, Faculty of Medicine, Erzincan Binali Yıldırım University, Erzincan 24100, Turkey; drcerenkarakurt@gmail.com (T.C.O.K.); drufuk2001@gmail.com (U.K.); 3 Department of Anesthesiology and Reanimation, Kovancılar State Hospital, Elazığ 23850, Turkey; talha1905-74@hotmail.com; 4 Department of Radiology, Faculty of Medicine, Erzincan Binali Yıldırım University, Erzincan 24100, Turkey; ilker.atas44@gmail.com

**Keywords:** anesthesia, pediatric, fasting, anxiety, ultrasonography, carbohydrates, gastric emptying

## Abstract

**Background/Objectives**: Shortened clear-fluid fasting is increasingly supported in pediatric anesthesia, yet its combined effects on child-centered perioperative outcomes, family satisfaction, metabolic response, and gastric ultrasonography findings remain unclear. This study evaluated the associations of two routine preoperative fasting practices with preoperative anxiety, perioperative blood glucose, hemodynamic parameters, family satisfaction, and gastric ultrasonography findings in children undergoing elective surgery. **Methods**: This prospective, two-arm, observational cohort study included 66 children aged 3–12 years with American Society of Anesthesiologists (ASA) physical status I–II scheduled for elective adenotonsillectomy under general anesthesia, following either standard overnight fasting or 5 mL/kg carbohydrate-containing clear apple juice approximately 2 h before surgery. Preoperative anxiety was assessed using the Modified Yale Preoperative Anxiety Scale; blood glucose, hemodynamic parameters, gastric antral cross-sectional area, estimated gastric residual volume, Perlas classification, family satisfaction, fasting duration, and nausea and vomiting were also evaluated. **Results**: Preoperative anxiety scores were significantly lower in the clear-fluid group than in the overnight fasting group (39.95 ± 20.02 vs. 51.88 ± 22.05; *p* = 0.003). No significant differences were observed between groups in blood glucose, gastric antral cross-sectional area, estimated gastric residual volume, or Perlas scores. Family satisfaction was significantly higher in the clear-fluid group (8.76 ± 1.66 vs. 4.12 ± 2.00; *p* < 0.001), and fasting duration was markedly shorter. No perioperative pulmonary aspiration occurred. **Conclusions**: Administration of 5 mL/kg of carbohydrate-containing clear fluid approximately 2 h before surgery was associated with lower preoperative anxiety and higher family satisfaction, with no significant between-group difference in gastric residual volume.

## 1. Introduction

Pulmonary aspiration is regarded as one of the most critical perioperative complications that can occur during general anesthesia. Since the suppression of protective airway reflexes increases the risk of regurgitation and aspiration of gastric contents, preoperative fasting protocols have become a fundamental safety component of anesthetic practice. Consequently, different fasting durations are recommended for solid foods, breast milk, and clear fluids; the goal is to minimize the risk of aspiration by allowing sufficient time for gastric emptying [1,2].

However, it is recognized that fasting periods implemented to improve safety, especially in pediatric patients, are often longer than recommended. Due to their physiological characteristics, children are more significantly affected by prolonged fasting than adults. Due to limited glycogen stores, a high basal metabolic rate, and a more fragile fluid balance, prolonged fasting periods can lead to hypoglycemia, dehydration, ketosis, and hemodynamic instability. Additionally, the combination of hunger and thirst with the hospital environment, separation from parents, anticipation of pain, and uncertainty can increase preoperative anxiety in children. These circumstances can negatively affect not only psychological comfort but also cooperation during anesthesia induction and the perioperative experience [3,4,5,6,7,8].

In recent years, more liberal clear-liquid fasting protocols have gained attention. It is known that clear fluids leave the stomach more rapidly than particulate or fatty contents. Consequently, current international guidelines state that clear-liquid intake may be permitted more flexibly during the preanesthetic period in appropriate patient groups. This approach aims to enhance patient comfort and physiological balance while maintaining aspiration safety [2,3].

It is believed that the administration of clear fluids in children prior to surgery not only reduces feelings of hunger and thirst but may also positively influence metabolic stability and perioperative hemodynamic trends. Ensuring adequate oral fluid intake during the preoperative period may also contribute to children being psychologically calmer and more cooperative. However, the number of studies that comprehensively evaluate this relationship, especially those addressing anxiety, metabolic stability, and aspiration safety together, is limited [5,6,7,8,9].

Gastric ultrasonography is a prominent noninvasive point-of-care imaging method for assessing the safety of preoperative fasting protocols. By evaluating the gastric antrum, information about gastric contents can be obtained, and gastric residual volume can be estimated. Thus, an individualized aspiration risk assessment can be performed based not only on the theoretical fasting duration but also on actual gastric contents. This methodology is particularly important for the safe evaluation of more flexible fasting strategies related to clear-fluid administration [2,10,11].

In pediatric surgery, physiological and psychological outcomes as well as family satisfaction are important. Keeping a child hungry and restless for an extended period can increase parental anxiety, which may also affect the child’s perioperative experience. Conversely, a more comfortable preoperative process can enhance family compliance and satisfaction. Therefore, when evaluating the outcomes of preoperative fasting protocols, the family’s experience should also be taken into account [4,9].

In this study, two different preoperative fasting approaches used in clinical practice were prospectively compared. Patients were grouped according to the preoperative fluid regimen administered, without random assignment by the researcher. In one group, patients were allowed to consume 5 mL/kg of clear fluids up to two hours before surgery, while the traditional fasting protocol was maintained in the other group. The main aim of the study was to compare these two routine fasting approaches with respect to preoperative anxiety, perioperative blood glucose levels, and gastric ultrasonography findings in children. Secondarily, hemodynamic parameters, family satisfaction, actual fasting durations, and preoperative/postoperative nausea and vomiting symptoms were examined. In this context, the study aimed to highlight the adverse effects of prolonged fasting periods and the clinical acceptability of current fasting protocols. A recent systematic review and meta-analysis of eight randomized controlled trials (1234 children) found no significant difference in gastric residual volume or gastric pH between shorter (≤1 h) and longer clear-fluid fasting regimens and no increased risk of pulmonary aspiration, while explicitly identifying metabolic and stress responses and the specific role of carbohydrate-containing fluids as priorities for future research [12]. However, individual studies to date, including those included in that meta-analysis, have generally examined these domains—gastric emptying, metabolic response, or fasting duration—in isolation rather than concurrently. The additional clinical value of the present study lies in the simultaneous assessment of child-centered outcomes, including preoperative anxiety and family satisfaction, together with perioperative glucose levels, hemodynamic trends, actual fasting duration, and gastric ultrasonography findings within the same routine-care pediatric cohort. This multidimensional approach was intended to evaluate both physiological outcomes and the clinical acceptability of shortened fasting practice.

## 2. Materials and Methods

This prospective, two-arm, observational cohort study was carried out between 20 May 2024, and 20 December 2024, in the operating room at Erzincan Binali Yıldırım University Mengücek Gazi Training and Research Hospital. The study assessed two preoperative fasting protocols utilized in standard perioperative clinical care for pediatric patients. The investigators did not assign the preoperative fasting regimen. Group classification reflected the fasting practice applied during routine clinical care by the attending anesthesia team in conjunction with the planned surgical schedule. No study-specific allocation criteria, randomization procedure, or investigator-directed assignment was used. Because these routine-care decisions may have been influenced by clinical workflow, scheduling characteristics, or clinician preferences that were not fully captured in the study dataset, residual confounding cannot be excluded.

**Figure 1:**
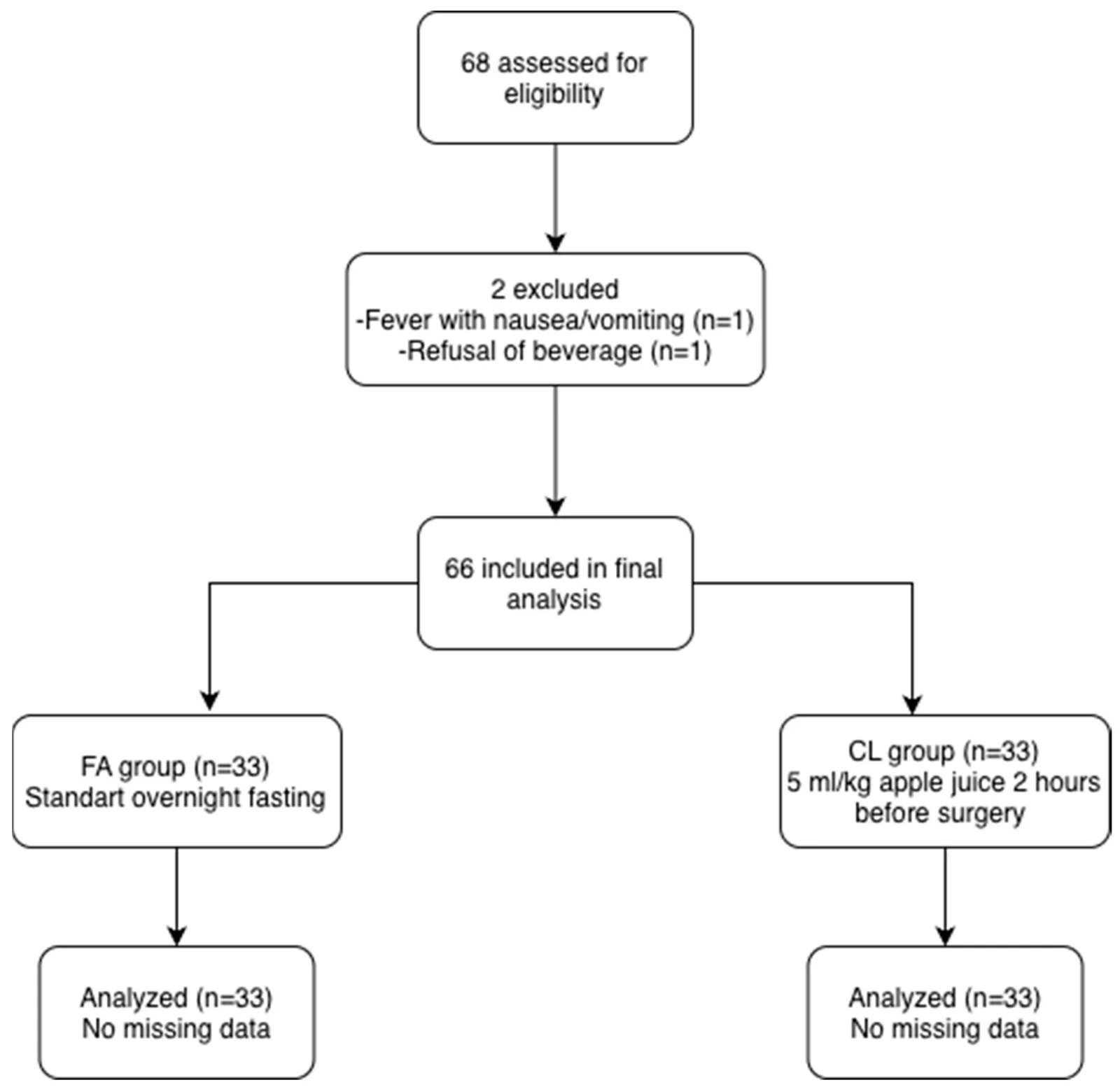
Flow diagram of patient inclusion and analysis.

Accordingly, the study was considered observational because the investigators prospectively recorded outcomes associated with fasting practices already used in routine clinical care rather than assigning the exposure. Approval from the Erzincan Binali Yıldırım University Clinical Research Ethics Committee (Decision No. 2024-07/07, dated 2 May 2024) was obtained. All participants’ parents or legal guardians provided written informed consent, and all procedures were carried out in compliance with the Declaration of Helsinki. The study was carried out in accordance with the Strengthening the Reporting of Observational Studies in Epidemiology (STROBE) guidelines, and patient inclusion is summarized in Figure 1, with a detailed study protocol available in Supplementary File S1.

Although data were prospectively collected from a heterogeneous pediatric surgical cohort, the present analysis was restricted to patients undergoing elective adenotonsillectomy in order to reduce procedural heterogeneity. For the present analysis, children aged 3–12 years with ASA physical status I–II who underwent elective adenotonsillectomy under general anesthesia were included. Exclusion criteria were emergency surgery, American Society of Anesthesiologists physical status III or higher, neurological disorders, gastrointestinal diseases potentially affecting gastric emptying, type 1 diabetes mellitus, liver or kidney disease, electrolyte imbalance, extreme obesity, advanced cachexia, known drug allergy, incapacity to cooperate with study assessments, or refusal to participate. Patients in the clear-fluid group received 5 mL/kg of commercially available 100% apple juice without added sugar, containing 11.5 g of carbohydrate per 100 mL, with administration targeted approximately 2 h before induction of anesthesia. Because the study reflected routine perioperative practice, the actual interval between clear-fluid intake and anesthesia induction varied according to clinical and operating-room workflow. The beverage was a commercially available apple juice rather than a standardized carbohydrate-loading solution.

Sample size was calculated a priori using G*Power software, version 3.1.9.7, for a two-tailed independent-samples *t*-test comparing preoperative m-YPAS scores between the two study groups. The calculation was informed by previous pediatric data with a similar design and outcome structure, and a conservative standardized effect size of Cohen’s d = 0.78 was used. With a type I error rate of 0.05, statistical power of 80%, and a 1:1 allocation ratio, the minimum required sample size was 54 patients (27 per group). The planned sample size was increased to 68 patients to allow for potential exclusions and data loss. Among the 68 patients initially assessed for eligibility, 2 patients were excluded before final analysis: one because of fever accompanied by nausea and vomiting and one because of refusal to consume the beverage after tasting it. Because the latter patient had already been exposed to the clear fluid, the patient was not reassigned to the overnight fasting group.

The primary outcomes of the study were preoperative anxiety, perioperative blood glucose levels, and the results of gastric ultrasonography (gastric antral cross-sectional area and estimated gastric residual volume). Because multiple primary outcomes representing different clinical domains were evaluated, the findings for individual outcomes should be interpreted in the context of the exploratory observational design. No formal adjustment for multiplicity across the primary outcomes was applied. Secondary outcomes included hemodynamic parameters, gastric ultrasound risk grading according to the Perlas classification, family satisfaction, actual fasting duration, and preoperative and postoperative nausea and vomiting symptoms. Family satisfaction and actual fasting duration were also evaluated to assess clinical acceptability of shorter clear-fluid fasting practices in routine pediatric perioperative care. Actual fasting duration was analyzed as observed, including deviations from the targeted 120 min interval.

The age, sex, height, weight and body mass index for each participant were recorded. Anesthesia duration was obtained from the anesthesia records and recorded for each patient for between-group comparison. Preoperative anxiety was assessed once in the preoperative waiting area before transfer to the operating room and before administration of anesthetic drugs using the Modified Yale Preoperative Anxiety Scale (mYPAS). All assessments were performed by the same investigator under the same routine preoperative conditions. The investigator performing the mYPAS assessment was blinded to the patient’s preoperative fasting group.

Family satisfaction was assessed preoperatively, before induction of anesthesia, using a researcher-developed, study-specific assessment. Parents in both groups were asked the same core satisfaction questions: whether they were satisfied with the fasting approach applied to their child (yes/no) and to provide a single overall satisfaction score from 1 to 10. In the clear-fluid group, 10 predefined aspects were used as structured anchors for the overall rating and were not analyzed as separate subscale scores. In addition, before answering the preference question, parents in the overnight fasting group were provided with a standardized face-to-face explanation of the two routine fasting approaches, including the alternative carbohydrate-containing clear-fluid approach. They were then asked whether they would have preferred the clear-fluid approach had it been available as an alternative (yes/no). This preference question was exploratory and did not influence the fasting regimen, perioperative management, or any clinical decision. The assessment was not formally validated; therefore, the findings should be considered exploratory and may be subject to measurement bias. A standardized representation of the assessment format is provided in Supplementary File S3.

Gastric ultrasonography was conducted after overnight fasting in the overnight fasting group and after clear-fluid intake, before induction of anesthesia, in the clear-fluid group. The same anesthesiologist, with experience in perioperative gastric ultrasonography, conducted all examinations. The anesthesiologist performing the gastric ultrasonography assessment was blinded to the patient’s preoperative fasting group. Ultrasound examinations were performed in the supine and right lateral decubitus positions utilizing a convex probe (1–8 MHz; AC2541; MyLab Six, Esaote S.p.A., Genova, Italy). The gastric antrum was identified in the sagittal plane between the left lobe of the liver and the pancreas at the level of the descending aorta and superior mesenteric artery. Anteroposterior (AP) and craniocaudal diameters of the gastric antrum were measured, and gastric antral cross-sectional area (CSA) was calculated as CSA = (AP × CC × π)/4. The right lateral decubitus CSA was used to estimate gastric residual volume according to the pediatric age-adjusted predictive model described by Spencer et al.: GRV (mL) = −7.8 + (3.5 × RLD CSA) + [0.127 × age (months)] [11].

All examinations were performed by the same anesthesiologist using the same patient positions, anatomical landmarks, and measurement protocol to minimize interobserver variability. Formal intraobserver reproducibility testing was not performed.

Estimated gastric residual volume was calculated using previously defined formulas based on gastric antral measurements and patient age. The Perlas grading system was also used for qualitative assessment of gastric fluid: grade 0 indicates no fluid visible in either the supine or right lateral decubitus position, grade 1 indicates fluid visible only in the right lateral decubitus position, and grade 2 indicates fluid visible in both positions [10]. When suitable aspiration precautions were required, a gastric residual volume of more than 1.5 mL/kg or the presence of solid or thick fluid content was deemed symptomatic of increased aspiration risk. For patients with an estimated gastric residual volume greater than 1.5 mL/kg, induction of anesthesia was delayed and an additional waiting period was allowed before proceeding.

Intraoperative monitoring included electrocardiography, peripheral oxygen saturation, and noninvasive blood pressure monitoring. No premedication was administered in the ward before transfer to the operating room. General anesthesia was induced with intravenous midazolam (0.05 mg/kg), fentanyl (1 µg/kg), propofol (2.5–3 mg/kg, titrated to clinical effect), and rocuronium bromide (0.6 mg/kg). Airway management was performed via endotracheal intubation. Anesthesia was maintained with sevoflurane in a combination of 50% oxygen and air, and intraoperative intravenous paracetamol (10 mg/kg) was given to patients without contraindications. No dedicated quantitative depth-of-anesthesia monitoring was used, and individual MAC values were not systematically recorded in the study dataset. Maintenance fluid therapy used isotonic saline solution dependent on body weight. All patients received a single intraoperative intravenous dose of ondansetron (0.15 mg/kg) for postoperative nausea and vomiting prophylaxis. No repeat dose of ondansetron or additional rescue antiemetic was administered during the evaluated perioperative period. Neuromuscular blockade was reversed using atropine and neostigmine at the end of surgery.

Hemodynamic monitoring was performed continuously throughout the perioperative period. To minimize repeated invasive sampling in pediatric patients, blood glucose concentrations were measured from capillary blood obtained by finger-prick using a FreeStyle Optium Neo H bedside glucometer (Abbott Diabetes Care Ltd., Witney, UK). Measurements were obtained at three predefined perioperative phases rather than at fixed clock times: preoperatively before induction of anesthesia, intraoperatively, and postoperatively in the recovery unit. The intraoperative measurement was targeted within the first hour of anesthesia; however, because anesthesia duration varied among patients, in shorter procedures it was obtained before completion of the procedure rather than at an exact 60 min time point. Blood or urinary ketone levels were not routinely measured as part of the study protocol. Hemodynamic variables were recorded at the same three perioperative phases for statistical analysis. The recorded hemodynamic parameters were systolic blood pressure, diastolic blood pressure, mean arterial pressure, and heart rate. Preoperative nausea and vomiting symptoms were assessed clinically before anesthesia induction. The Baxter Animated Retching Faces (BARF) scale, a six-face pictorial nausea scale scored from 0 (no nausea) to 10 (maximum nausea/retching), was used in the recovery unit to assess postoperative nausea intensity as part of the postoperative nausea and vomiting assessment; symptoms were further tracked during early ward follow-up [13]. Patients were sent to the ward after receiving an Aldrete score of 9 or higher.

Selection bias and residual confounding cannot be completely excluded as patients were followed up using standard clinical fasting procedures instead of randomized allocation. Age, perioperative stress response, baseline anxiety features, and individual clinical heterogeneity were all potential confounding factors. To reduce heterogeneity, only patients with elective American Society of Anesthesiologists physical status I–II within a comparable age range were included, and all evaluations were carried out in the same clinical setting based on a standardized perioperative protocol.

Statistical analyses were performed using IBM SPSS Statistics for Mac, version 25.0 (IBM Corp., Armonk, NY, USA). Distribution normality was assessed using the Shapiro–Wilk test. Continuous variables are presented as mean ± standard deviation or median (minimum–maximum), according to data distribution, whereas categorical variables are presented as number (%). Comparisons between groups were performed using the independent-samples *t*-test or Mann–Whitney U test for continuous variables and Pearson’s chi-square test for categorical variables, as appropriate. Relationships between continuous variables were evaluated using Spearman correlation analysis. As an exploratory analysis, associations between actual fasting duration and the primary outcomes were assessed using Spearman correlation coefficients. An exploratory repeated-measures analysis of variance was additionally performed to assess group × time interactions for perioperative blood glucose and hemodynamic parameters measured at the three predefined time points.

## 3. Results

The eligibility of 68 pediatric patients between 20 May 2024, and 20 December 2024, was evaluated. Two patients were excluded before final analysis: one because of fever accompanied by nausea and vomiting and one because of refusal to consume the beverage. As a result, 66 patients, 33 in each group, were included in the final analysis and there were no missing data. Figure 1 summarizes patient inclusion and analysis.

Demographic characteristics (as shown in Table 1) were similar between groups, as age, sex distribution, height, weight, and body mass index did not show any statistically significant differences.

Anesthesia duration was significantly longer in the overnight fasting group than in the clear-fluid group (92.03 ± 22.93 min vs. 72.55 ± 19.20 min; median [minimum–maximum], 90 [55–170] min vs. 80 [40–110] min; *p* < 0.001). The available perioperative records did not allow the specific reasons for this between-group difference to be determined. 

**Figure 2:**
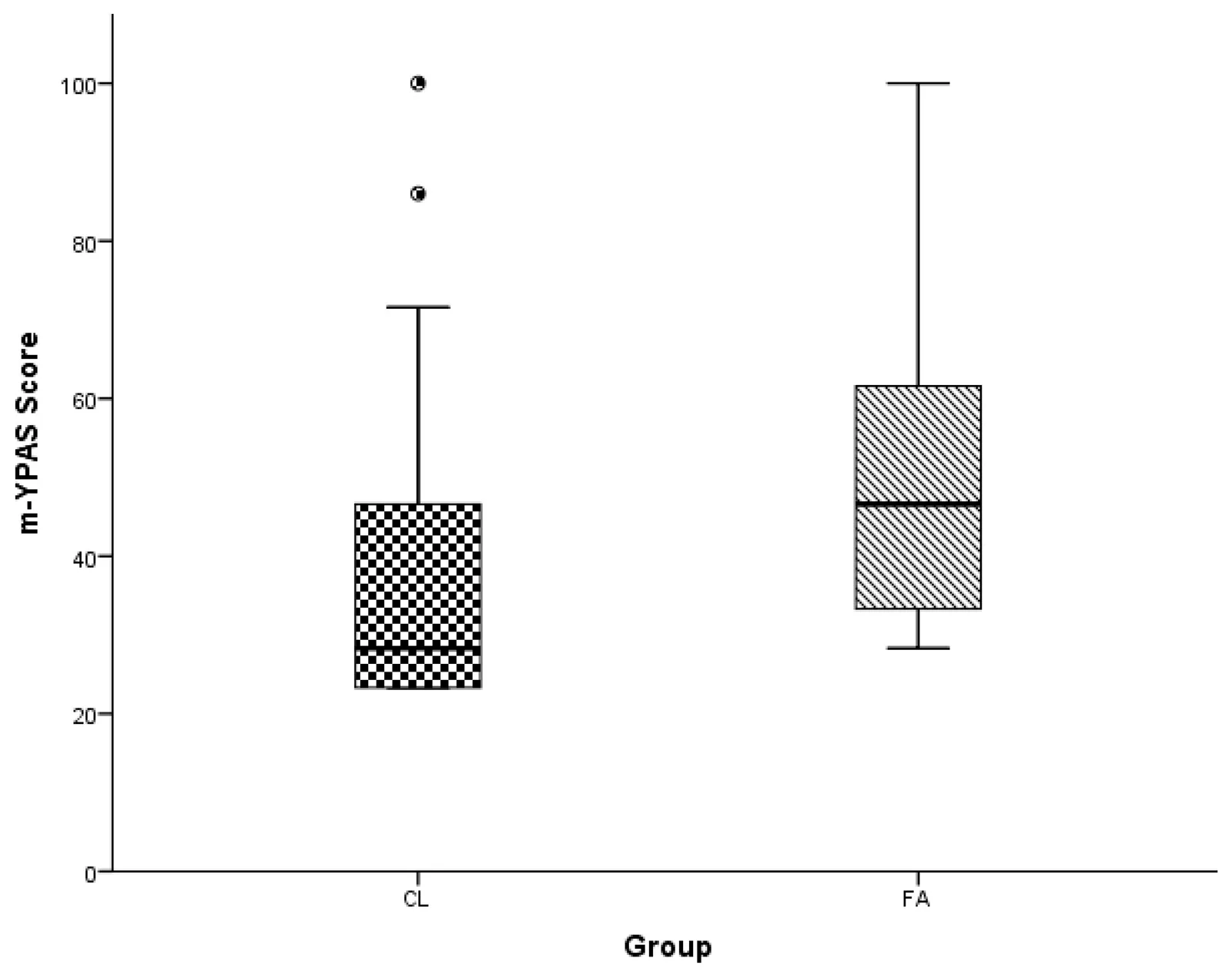
Distribution of preoperative Modified Yale Preoperative Anxiety Scale (m-YPAS) scores between the clear-fluid group and the overnight fasting group. The boxes represent the interquartile ranges (25th–75th percentiles), the horizontal lines within the boxes indicate the medians, the whiskers extend to the most extreme values within 1.5 times the interquartile range, and the circles indicate outliers.

Preoperative m-YPAS scores were significantly lower in the clear-fluid group than in the overnight fasting group (51.88 ± 22.05 vs. 39.95 ± 20.02; median [min–max], 46.6 [28.3–100] vs. 28.3 [23.3–100]; *p* = 0.003), as shown in Table 2 and Figure 2. In an exploratory item-level analysis of the mYPAS, scores were lower in the clear-fluid group for activity (1.52 ± 0.87 vs. 1.91 ± 0.98; *p* = 0.042), vocalization (2.03 ± 1.72 vs. 2.52 ± 1.70; *p* = 0.036), emotional expressivity (1.97 ± 0.98 vs. 2.61 ± 0.83; *p* = 0.008), and state of apparent arousal (1.45 ± 0.83 vs. 2.15 ± 1.12; *p* = 0.005). No significant between-group difference was observed for use of parents (1.70 ± 0.81 vs. 2.03 ± 1.05; *p* = 0.222). These item-level analyses were exploratory.

**Figure 3:**
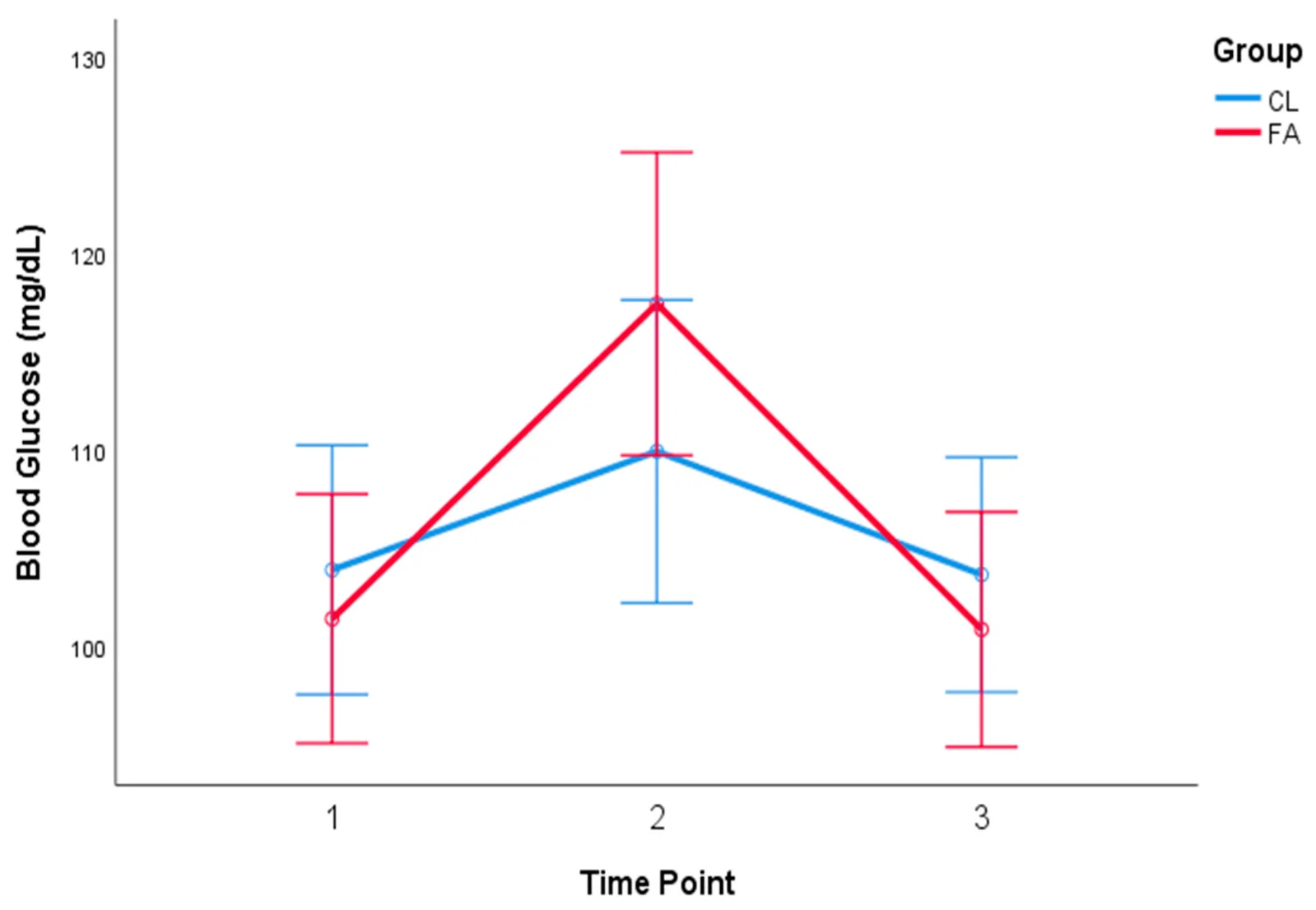
Perioperative blood glucose changes over time in the clear-fluid and overnight fasting groups. Time point 1: preoperative measurement; time point 2: intraoperative measurement; time point 3: postoperative measurement.

No statistically significant differences were observed between groups in perioperative blood glucose measurements at any predefined time point. Blood glucose measurements are presented in Table 3, while perioperative blood glucose changes over time are illustrated in Figure 3.

No statistically significant differences were noted among the groups for gastric antral cross-sectional area, estimated gastric residual volume, or Perlas classification scores. These gastric ultrasonography findings are summarized in Table 4.

Estimated gastric residual volume exceeded 1.5 mL/kg in one patient in the clear-fluid group and in two patients in the overnight fasting group. In these three patients, induction of anesthesia was delayed and an additional waiting period was allowed before proceeding. No perioperative pulmonary aspiration event was observed in these patients; however, this descriptive observation should not be interpreted as evidence of equivalent aspiration safety between the fasting approaches.

No statistically significant differences were observed in hemodynamic measurements such as systolic blood pressure, diastolic blood pressure, or mean arterial pressure between groups at any predefined time point, as shown in Table 5. Heart rate measurements were similar between groups during the first and second measurements but were lower in the clear-fluid group during the postoperative measurement period (*p* < 0.001).

An exploratory repeated-measures analysis revealed no significant group × time interaction for blood glucose, diastolic blood pressure, mean arterial pressure, or heart rate measurements (all *p* > 0.05). However, systolic blood pressure showed a significant group × time interaction (*p* = 0.025), with greater fluctuation in the overnight fasting group; blood glucose measurements showed a similar trend that did not reach significance (interaction *p* = 0.062).

**Figure 4:**
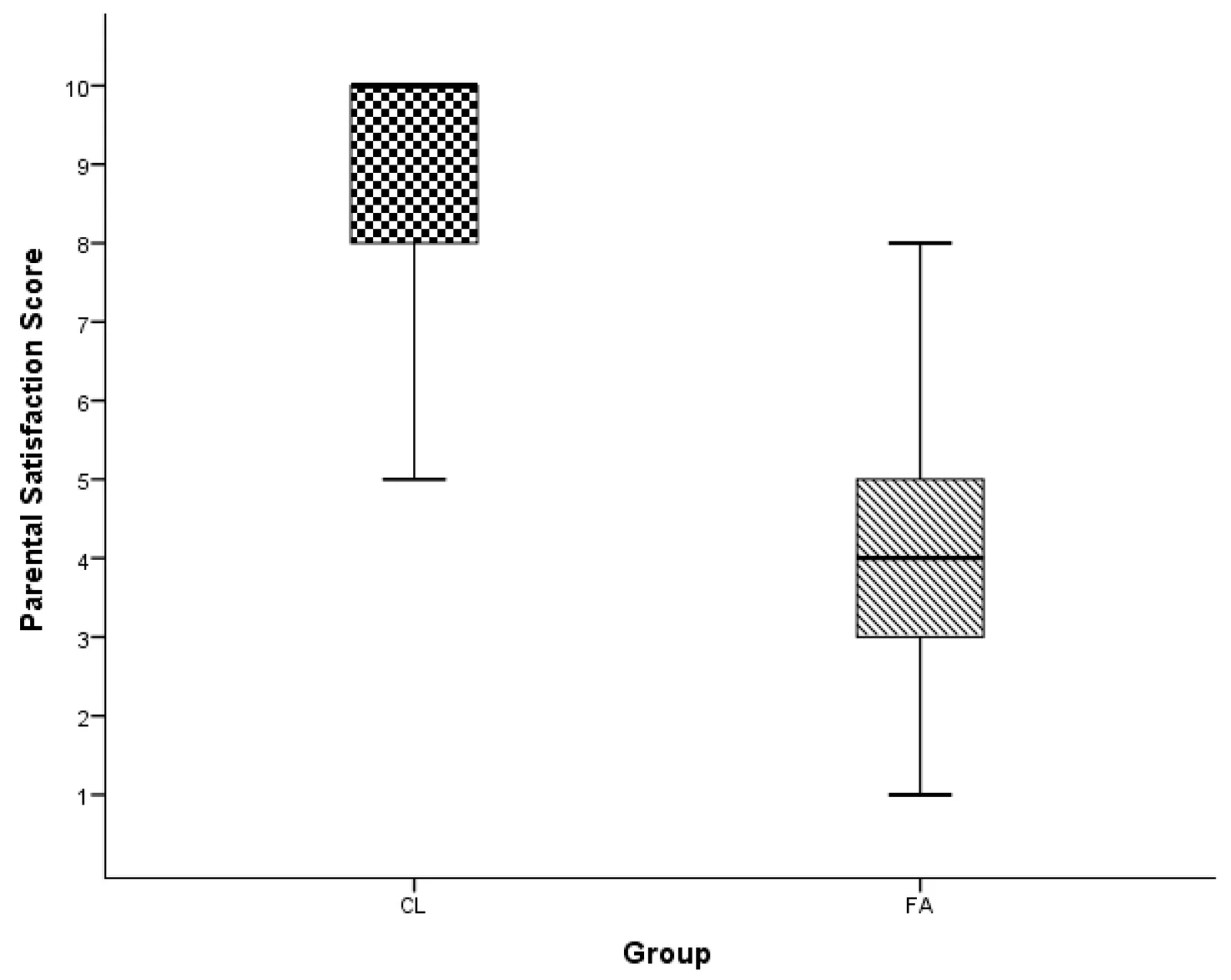
Parental satisfaction scores between the clear-fluid group and the overnight fasting group. The boxes represent the interquartile ranges (25th–75th percentiles), the horizontal lines within the boxes indicate the medians, the whiskers extend to the most extreme values within 1.5 times the interquartile range, and the circles indicate outliers.

Family satisfaction scores, as shown in Figure 4, were significantly higher in the clear-fluid group than in the overnight fasting group (8.76 ± 1.66 vs. 4.12 ± 2.00; *p* < 0.001). When parents in the overnight fasting group were asked whether they would have preferred the carbohydrate-containing clear-fluid approach had it been available, all 33 parents (100%) responded affirmatively. This preference was expressed after parents had received the standardized explanation of the alternative fasting approach. Because the additional structured components of the satisfaction assessment differed between groups, the between-group comparison of the overall satisfaction score should be interpreted as exploratory. Observed actual fasting duration was 619.39 ± 120.31 min in the overnight fasting group and 115.45 ± 22.93 min in the clear-fluid group.

In the clear-fluid group, actual fasting duration ranged from 70 to 160 min; 15 of 33 children had an interval of less than 120 min, 8 had an interval of exactly 120 min, and 10 had an interval greater than 120 min. Thus, the intended 2 h timing was not achieved exactly in all patients.

In exploratory correlation analyses, longer actual fasting duration was associated with higher preoperative m-YPAS scores (Spearman’s ρ = 0.395; *p* = 0.001). Actual fasting duration was not significantly correlated with preoperative, intraoperative, or postoperative blood glucose levels (ρ = −0.001, *p* = 0.993, and ρ = 0.121; *p* = 0.333, ρ = −0.094, and *p*= 0.453, respectively), gastric antral CSA (ρ = 0.028; *p* = 0.822), or estimated GRV (ρ = −0.0004; *p* = 0.998).

No significant differences were observed between groups regarding preoperative or postoperative nausea and vomiting symptoms. In the recovery unit, there was no postoperative nausea or vomiting. One patient from each group experienced prolonged nausea and vomiting with delayed oral intake throughout ward follow-up after adenotonsillectomy.

## 4. Discussion

In this prospective observational study, administration of 5 mL/kg of a carbohydrate-containing clear fluid 2 h before surgery was associated with lower preoperative anxiety and higher family satisfaction in children undergoing elective surgery. Perioperative blood glucose measurements and gastric ultrasonography findings, including estimated gastric residual volume and gastric antral cross-sectional area, did not differ significantly between groups, and no perioperative pulmonary aspiration events were observed. Importantly, because group allocation was not randomized, the observed differences in anxiety and family satisfaction should be interpreted as associations rather than causal effects of carbohydrate-containing clear-fluid administration. An additional limitation is that the clear-fluid exposure combined two components: a substantially shorter fasting duration and intake of a commercially available carbohydrate-containing apple juice. Because the study did not include separate water-only, standardized carbohydrate-solution, or matched-fasting comparator groups, the observed associations cannot be attributed specifically to carbohydrate intake. They may reflect the effects of shorter fasting, fluid intake itself, carbohydrate content, or a combination of these factors. Accordingly, the present findings should be interpreted as associations with the overall clear-fluid fasting strategy rather than as evidence of a specific carbohydrate effect. The main contribution of this study is not the isolated demonstration of gastric emptying after clear-fluid intake, which has been addressed in previous pediatric fasting studies, but the simultaneous evaluation of psychological, metabolic, hemodynamic, family-centered, and gastric ultrasonography outcomes in a routine clinical pediatric cohort.

The lower preoperative anxiety scores observed in the clear-fluid group suggest an association between the overall clear-fluid fasting strategy and greater psychological comfort during the preoperative period. However, because fasting practice was not randomly assigned, the observed difference in preoperative anxiety cannot be attributed solely to clear-fluid intake or shorter fasting duration. Routine-care factors related to the attending anesthesia team, surgical scheduling, clinical workflow, or other unmeasured patient- or family-level characteristics may have influenced both group classification and preoperative anxiety. Therefore, a causal effect of carbohydrate-containing clear-fluid administration on preoperative anxiety cannot be inferred from the present study. Increased preoperative anxiety in pediatric patients has been associated with adverse perioperative and postoperative behavioral outcomes, and several nonpharmacological strategies have been shown to reduce anxiety in children [14,15,16,17,18,19]. This finding is consistent with a Turkish randomized trial that similarly reported the lowest anxiety scores and highest parental satisfaction in a carbohydrate-containing clear-fluid group compared with water or longer fasting [20]. The exploratory positive correlation between actual fasting duration and m-YPAS scores should nevertheless be interpreted cautiously because fasting duration was closely linked to study-group membership and may therefore reflect the same underlying confounding structure.

Perioperative blood glucose tests did not show any statistically significant changes between the groups. This result is in line with earlier research showing that shorter clear-fluid fasting regimens may not always result in significant changes in the perioperative glycemic responses of pediatric patients [21,22]. Perioperative glucose regulation is influenced by multiple factors, including surgical stress response, age, fluid therapy, and individual metabolic variability. Therefore, the absence of significant differences in blood glucose measurements between groups should be interpreted within the context of the multifactorial nature of perioperative metabolic regulation.

Perlas classification ratings, estimated gastric residual volume, and gastric antral cross-sectional area did not significantly differ across the groups. No statistically significant between-group differences were observed in Perlas classification, estimated gastric residual volume, or gastric antral cross-sectional area. However, the absence of statistically significant differences should not be interpreted as evidence of equivalence or equivalent aspiration safety, particularly given the limited sample size and the fact that the study was not designed or powered as an equivalence or safety trial. Earlier pediatric studies evaluating shortened clear-fluid fasting protocols using gastric ultrasonography have similarly reported gastric residual volume findings after clear-fluid intake [23,24]. These findings are also consistent with a recent large-scale meta-analysis of eight randomized controlled trials, which found no significant difference in gastric residual volume or gastric pH, and no increased risk of pulmonary aspiration, between shorter and longer clear-fluid fasting regimens in children [12]. In our study, gastric residual volume exceeded 1.5 mL/kg in a limited number of patients across both groups, further supporting the importance of personalized perioperative aspiration risk assessment rather than depending only on fasting duration. No perioperative pulmonary aspiration events were observed during the study period. However, because pulmonary aspiration is a rare event and the sample size was limited, this study was not powered to establish aspiration safety. Therefore, the absence of observed aspiration events should be interpreted descriptively and should not be considered evidence that the shortened clear-fluid fasting approach eliminates or does not increase aspiration risk.

There were no significant differences between groups in assessments of systolic, diastolic, or mean arterial pressure measurements. During the postoperative measurement period, the clear-fluid group had a lower heart rate, but no consistent differences were observed in other hemodynamic parameters. Previous studies of fasting protocols in children have demonstrated conflicting results about perioperative hemodynamic effects [5,21,22]. Perioperative hemodynamic responses are affected by multiple factors, such as anesthetic management, surgical stress response, intravascular volume status, and individual physiological variability. Intraoperative blood glucose levels may also be influenced by the surgical stress response and associated counter-regulatory hormonal changes; therefore, they cannot be considered a specific indicator of the metabolic effect of the preoperative fasting approach. As a result, the hemodynamic results of this study must be interpreted with caution. Exploratory repeated-measures analysis showed a statistically significant group × time interaction for systolic blood pressure (*p* = 0.025), whereas no significant group × time interactions were observed for the other hemodynamic parameters or blood glucose. Given the exploratory nature of these analyses and the difference in anesthesia duration between groups, these findings should be interpreted cautiously and should not be considered causal effects of the fasting regimen. Because anesthesia duration differed between groups and may independently influence perioperative glucose regulation and hemodynamic responses, the between-group findings for these outcomes remain potentially confounded. Accordingly, they should be interpreted as descriptive and exploratory rather than as causal effects of the fasting regimen.

Higher family satisfaction scores were observed in the clear-fluid group; however, this association should not be interpreted as evidence that shorter fasting itself caused greater satisfaction. Unmeasured parental characteristics, expectations, perioperative communication, clinician preferences, and scheduling factors may also have contributed to the observed difference. Although the same overall 1–10 satisfaction score was obtained in both groups, the additional assessment structure differed between groups; therefore, the magnitude of the observed between-group difference should be interpreted cautiously. Earlier quality-improvement studies have similarly reported improved comfort and reduced perioperative distress with shortened fasting durations in pediatric patients [25,26]. The potential relevance of improved preoperative comfort may extend beyond the immediate preoperative period. Preoperative anxiety has been associated with postoperative behavioral and recovery-related outcomes; however, the present study did not evaluate whether lower anxiety or greater family satisfaction was accompanied by differences in postoperative nursing care needs, recovery trajectory, or hospital length of stay. Future studies should therefore examine whether reduced preoperative anxiety is associated with postoperative nursing care requirements and recovery outcomes, including length of stay, while accounting for differences in pediatric medical and nursing care complexity. Recent work has emphasized that pediatric care complexity cannot be inferred solely from medical complexity and that standardized nursing data may provide additional information regarding care needs and hospital outcomes [27]. In face-to-face interviews conducted with clear-fluid group parents who gave low satisfaction ratings, the main reasons cited were the child’s dislike of the apple juice flavor and one parent’s reluctance to administer the clear fluid because of aspiration concerns based on information obtained outside the clinical team.

No nausea or vomiting was observed preoperatively in either group. Postoperatively, nausea and vomiting occurred in 1 of 33 patients (3.0%) in each group, several hours after surgery on the ward. There was no significant between-group difference in the incidence of nausea and vomiting.

Previous studies have suggested that postoperative nausea and vomiting in pediatric patients may be affected not only by fasting duration but also by surgical type and individual clinical factors [28]. The low number of nausea and vomiting events in the present study limits definitive interpretation of these findings. Anesthesia duration was significantly longer in the overnight fasting group despite restriction of the present analysis to children undergoing adenotonsillectomy. The specific reasons for this difference could not be determined from the available study data. Although all patients underwent the same general type of procedure, unmeasured differences in case complexity, anesthetic workflow, or other perioperative factors may have contributed; therefore, no specific explanation can be established from the present dataset. Importantly, anesthesia duration may have influenced intraoperative and postoperative physiological measurements and should be considered when interpreting blood glucose, hemodynamic, and postoperative outcomes. In contrast, preoperative anxiety and family satisfaction were assessed before induction of anesthesia; therefore, the subsequently observed difference in anesthesia duration cannot temporally account for these preoperative outcomes.

The current study’s results contribute to the growing evidence regarding shortened clear-fluid fasting regimens and perioperative comfort in pediatric patients, while no statistically significant between-group differences were observed in the evaluated gastric ultrasonography parameters. Reducing needlessly prolonged fasting periods may provide psychological advantages as well as support a more child- and family-centered approach to perioperative care. However, proper patient selection, unambiguous parental instructions, and well-coordinated perioperative organization are necessary for the implementation of shortened fasting regimens. When aspiration risk evaluation is clinically necessary, gastric ultrasonography may also be a helpful bedside technique for some patients.

First, the non-randomized group allocation represents a major limitation of this study. Patients followed routine clinical fasting practices rather than being randomly assigned to the study groups; therefore, selection bias and residual confounding cannot be excluded. Parental characteristics, clinician preferences, and scheduling practices may have influenced preoperative anxiety and family satisfaction independently of the fasting regimen. Consequently, the observed associations cannot establish a causal relationship between carbohydrate-containing clear-fluid administration and the reported child- and family-centered outcomes. Furthermore, the intervention combined shorter fasting with intake of carbohydrate-containing apple juice. Because no water-only or standardized carbohydrate-solution comparator was included, the independent contribution of carbohydrate intake cannot be distinguished from the effects of shorter fasting or fluid intake.

Second, the sample size was small and from a single center, potentially limiting generalizability. Third, the repeated-measures analysis was exploratory in nature, and the sample size was not specifically calculated for longitudinal analyses. Although realized anesthesia duration differed between groups in the overall cohort, both m-YPAS and family satisfaction were assessed before induction of anesthesia and therefore preceded anesthesia duration. Accordingly, anesthesia duration was not included as a covariate for these preoperative outcomes, although it may serve as a marker of underlying procedural complexity. Furthermore, perioperative blood glucose, hemodynamic parameters, and PONV were not adjusted for anesthesia duration. Therefore, between-group comparisons of these outcomes remain potentially confounded and should be regarded as descriptive and exploratory. The 2 h interval for clear-fluid intake represented the intended clinical timing rather than an exact interval achieved in every patient. Actual fasting duration varied with routine perioperative workflow, and 15 children in the clear-fluid group underwent anesthesia induction less than 120 min after their last clear-fluid intake. This variability should be considered when interpreting the findings. In addition, structured rating anchors were provided only for the clear-fluid group, which may have introduced differential measurement bias and limits the direct comparability of satisfaction scores between groups. Finally, family satisfaction was assessed using a study-specific, non-validated assessment. Consequently, its construct validity and reliability are unknown, and response or measurement bias cannot be excluded. Because this preference question was asked after explanation of the alternative approach, the information provided may itself have influenced parental responses and the 100% preference finding should therefore be interpreted descriptively. Therefore, the magnitude of the observed between-group difference in family satisfaction should be interpreted cautiously and considered exploratory until confirmed using validated parental satisfaction instruments.

## 5. Conclusions

In the present cohort of pediatric patients, the overall clear-fluid fasting strategy was associated with lower preoperative anxiety and higher family satisfaction, while no statistically significant between-group difference was observed in gastric residual volume. These findings support further investigation of shortened carbohydrate-containing clear-fluid fasting strategies in relation to perioperative comfort in children. To properly evaluate the metabolic and clinical consequences of shortened clear-fluid fasting protocols in pediatric perioperative care, more multicenter trials with larger sample sizes are necessary. Given the observational and non-randomized design, these findings should be interpreted as associations and do not establish causality. Between-group comparisons of perioperative blood glucose, hemodynamic parameters, and PONV should likewise be interpreted as descriptive and exploratory because they were not adjusted for differences in anesthesia duration. Importantly, the absence of pulmonary aspiration events in this cohort does not establish the safety of the protocol with respect to this rare outcome.

## Figures and Tables

**Table 1:** Baseline demographic characteristics of the study groups.

	Group FA (n = 33)	Group CL (n = 33)	*p* Value
**Age (months)**			
Mean ± SD	88.79 ± 31.94	82.12 ± 29.17	0.379
**Sex**			
Female	13 (39.4%)	12 (36.4%)	
Male	20 (60.6%)	21 (63.6%)	
**Height (m)**	1.19 ± 0.17	1.20 ± 0.15	0.774
**Weight (kg)**	26.68 ± 12.08	24.17 ± 9.73	0.430
**BMI (kg/m^2^)**	18.28 ± 5.14	16.38 ± 3.97	0.094

Data are presented as mean ± standard deviation or number (%). BMI, body mass index; CL, clear-fluid group; FA, overnight fasting group.

**Table 2:** m-YPAS Scores for the Groups.

m-YPAS Scores by Group	Group FA (n = 33)	Group CL (n = 33)	*p*-Value
**Preoperative Waiting Area m-YPAS**			
Mean ± SD	51.88 ± 22.05	39.95 ± 20.02	0.003
Median (Min–Max)	46.6 (28.3–100)	28.3 (23.3–100)	

Data are presented as mean ± standard deviation and median (minimum–maximum). m-YPAS, Modified Yale Preoperative Anxiety Scale; CL, clear-fluid group; FA, overnight fasting group.

**Table 3:** Perioperative blood glucose measurements in different study groups.

Blood Glucose Measurements	Group FA (n = 33)	Group CL (n = 33)	*p* Value
**Preoperative blood glucose**			
Mean ± SD	101.18 ± 15.54	103.61 ± 18.75	0.569
Median (min–max)	100 (72–127)	102 (74–140)	
**Intraoperative blood glucose**			
Mean ± SD	116.03 ± 21.35	108.88 ± 19.68	0.162
Median (min–max)	122 (76–155)	110 (71–142)	
**Postoperative blood glucose**			
Mean ± SD	100.45 ± 15.24	103.03 ± 16.33	0.351
Median (min–max)	99 (74–150)	103 (70–130)	

Data are presented as mean ± standard deviation and median (minimum–maximum). CL, clear-fluid group; FA, overnight fasting group.

**Table 4:** Gastric ultrasonography findings in different study groups.

Antral Area Measurements and Perlas Scores by Group	Group FA (n = 33)	Group CL (n = 33)	*p* Value
**Gastric antral cross-sectional area**			
Mean ± SD	3.81 ± 2.36	3.68 ± 2.49	0.590
Median (min–max)	2.85 (1.16–10.24)	3.07 (1.12–10.74)	
**Gastric residual volume**			
Mean ± SD	16.80 ± 10.49	15.50 ± 9.96	0.691
Median (min–max)	14.42 (3.58–45.57)	13.91 (1.45–44.52)	
**Perlas score**			
Mean ± SD	0.27 ± 0.57	0.27 ± 0.51	0.840
Median (min–max)	0 (0–2)	0 (0–2)	

Data are presented as mean ± standard deviation and median (minimum–maximum). CL, clear-fluid group; FA, overnight fasting group.

**Table 5:** Perioperative hemodynamic measurements in different study groups.

Hemodynamic Parameters	Group FA (n = 33)	Group CL (n = 33)	*p* Value
**Preoperative SBP**			
Mean ± SD	104.52 ± 10.43	100.30 ± 10.15	0.101
Median (min–max)	102 (86–124)	100 (80–121)	
**Intraoperative SBP**			
Mean ± SD	100.55 ± 10.53	100.06 ± 8.30	0.872
Median (min–max)	98 (78–123)	100 (85–115)	
**Postoperative SBP**			
Mean ± SD	101.67 ± 8.24	103.48 ± 7.38	0.349
Median (min–max)	100 (82–120)	102 (88–120)	
**Preoperative DBP**			
Mean ± SD	61.88 ± 10.71	58.15 ± 7.95	0.114
Median (min–max)	62 (43–89)	59 (41–72)	
**Intraoperative DBP**			
Mean ± SD	58.21 ± 10.31	58.45 ± 7.21	0.630
Median (min–max)	56 (40–83)	60 (48–76)	
**Postoperative DBP**			
Mean ± SD	63.03 ± 8.63	62.06 ± 6.68	0.612
Median (min–max)	63 (50–80)	63 (50–76)	
**Preoperative MAP**			
Mean ± SD	76.09 ± 9.95	72.20 ± 7.76	0.082
Median (min–max)	74.67 (58.67–100)	72 (60–85.67)	
**Intraoperative MAP**			
Mean ± SD	72.32 ± 9.24	72.32 ± 6.29	0.663
Median (min–max)	70.67 (58.67–94.67)	71.67 (61.33–84)	
**Postoperative MAP**			
Mean ± SD	75.91 ± 7.83	75.87 ± 6.16	0.981
Median (min–max)	74.67 (62–90)	76.67 (65.33–86.67)	
**Preoperative HR**			
Mean ± SD	109.06 ± 16.76	103.67 ± 13.70	0.157
Median (min–max)	110 (80–152)	101 (80–132)	
**Intraoperative HR**			
Mean ± SD	106.30 ± 16.27	103.15 ± 15.80	0.428
Median (min–max)	108 (68–143)	102 (72–146)	
**Postoperative HR**			
Mean ± SD	107.42 ± 11.59	97.30 ± 10.77	<0.001
Median (min–max)	110 (85–126)	98 (75–120)	

Data are presented as mean ± standard deviation and median (minimum–maximum). CL, clear-fluid group; DBP, diastolic blood pressure; FA, overnight fasting group; HR, heart rate; MAP, mean arterial pressure; SBP, systolic blood pressure.

## Data Availability

The data presented in this study are available upon request from the corresponding author due to privacy and ethical restrictions.

## Supplementary Materials

The following supporting information can be downloaded at: https://www.mdpi.com/article/10.3390/nu18213684/s1, Supplementary File S1: Study protocol; Supplementary File S2: STROBE checklist; Supplementary File S3: Family satisfaction assessment.

## Abbreviations

The following abbreviations are used in this manuscript:

ASA	American Society of Anesthesiologists
BARF	Baxter Animated Retching Faces
BMI	Body Mass Index
CL	Clear-Fluid Group
CSA	Cross-Sectional Area
DBP	Diastolic Blood Pressure
FA	Overnight Fasting Group
GRV	Gastric Residual Volume
HR	Heart Rate
MAP	Mean Arterial Pressure
mYPAS	Modified Yale Preoperative Anxiety Scale
SBP	Systolic Blood Pressure
STROBE	Strengthening the Reporting of Observational Studies in Epidemiology
