# Supplementary material for: Metabolic, Gastric, and Psychosocial Correlates of Carbohydrate-Containing Clear-Fluid Intake in Children Undergoing Adenotonsillectomy: A Prospective Cohort Study
Source: Nutrients. 2026 Nov 6;18(21):3684. doi: 10.3390/nu18213684 (PMC13765679; doi:10.3390/nu18213684)
Supplement: Supplementary file 1 [file nutrients-18-03684-s001.zip › Supplementary File S1- Study Protocol.pdf]

SUPPLEMENTARY MATERIALS 1

STUDY PROTOCOL

Metabolic, Gastric, and Psychosocial Correlates of Carbohydrate-Containing Clear-Fluid Intake in Children Undergoing Adenotonsillectomy: A Prospective Cohort Study

STUDY DESIGN

This study was designed as a prospective observational cohort study conducted in pediatric patients undergoing elective surgery under general anesthesia. Data were prospectively collected from a heterogeneous pediatric surgical cohort. For the present analysis, the cohort was restricted to patients undergoing elective adenotonsillectomy in order to reduce procedural heterogeneity. No intervention was assigned by the investigators, and patients were observed according to routine clinical practice.

STUDY SETTING

The study was conducted in the operating room of Erzincan Binali Yıldırım University Mengücek Gazi Training and Research Hospital between May 20, 2024 and December 20, 2024.

Participants

For the present analysis, children aged 3–12 years with American Society of Anesthesiologists (ASA) physical status I–II who underwent elective adenotonsillectomy under general anesthesia were included.

Exclusion Criteria

Patients undergoing emergency surgery, those classified as ASA physical status III or higher, patients with neurological disorders, gastrointestinal diseases affecting gastric emptying, type 1 diabetes mellitus, liver and/or kidney disease, electrolyte imbalance, severe obesity, advanced cachexia, known drug allergy, refusal to participate, or inability to cooperate with study procedures were excluded.

Study Groups

The study was conducted according to routine clinical practice. No intervention was assigned by the investigators. Participants were categorized according to the fasting regimen they received as part of routine clinical care.

• Fasting Group (FA): Patients who underwent conventional overnight fasting.

• Clear-Fluid Group (CL): Patients who received 5 mL/kg carbohydrate-containing clear apple juice approximately 2 hours before surgery.

STUDY OUTCOMES

Primary Outcomes

The primary outcomes were preoperative anxiety level, perioperative blood glucose measurements, gastric antral cross-sectional area (CSA), and estimated gastric residual volume (GRV). Preoperative anxiety was assessed using the Modified Yale Preoperative Anxiety Scale (m-YPAS).**Secondary Outcomes**

Secondary outcomes included Perlas classification, perioperative hemodynamic parameters, family satisfaction, actual fasting duration, and preoperative and postoperative nausea and vomiting.**Sample Size Calculation**

Sample size estimation was performed using G*Power software. Assuming a type I error rate of 0.05 and a statistical power of 80%, the minimum required sample size was calculated as 54 patients. To compensate for potential exclusions and data loss, the planned sample size was increased to 68 patients.

Data Collection and Measurements

Demographic characteristics, including age, sex, height, weight, and body mass index, were recorded for all participants.

Gastric ultrasonography was performed by an anesthesiologist experienced in perioperative gastric ultrasound. Gastric antral cross-sectional area and estimated gastric residual volume were calculated using previously published methods. Gastric contents were additionally classified according to the Perlas grading system.

Blood glucose measurements were obtained at three predefined perioperative phases: preoperatively before induction of anesthesia, intraoperatively within the first hour of anesthesia when feasible, and postoperatively in the recovery unit.

Perioperative hemodynamic parameters, preoperative and postoperative nausea and vomiting were also recorded.

Statistical Analysis

Statistical analyses were performed using IBM SPSS Statistics version 25.0 (IBM Corp., Armonk, NY, USA). Continuous variables were assessed for normality using the Shapiro–Wilk test. Group comparisons were performed using the independent-samples t-test or Mann–Whitney U test, as appropriate. Categorical variables were compared using Pearson's chi-square test. A two-sided p value < 0.05 was considered statistically significant. In addition, an exploratory repeated-measures analysis of variance was performed to assess time effects and group × time interactions for perioperative blood glucose and hemodynamic measurements.

Ethics Approval

The study was approved by the Erzincan Binali Yıldırım University Clinical Research Ethics Committee (Decision No. 2024-07/07, dated May 2, 2024). Written informed consent was obtained from the parents or legal guardians of all participants before enrollment.

Reporting Guideline

This study was conducted as an observational study and was reported in accordance with the Strengthening the Reporting of Observational Studies in Epidemiology (STROBE) guidelines.
