# Supplementary material for: Metabolic, Gastric, and Psychosocial Correlates of Carbohydrate-Containing Clear-Fluid Intake in Children Undergoing Adenotonsillectomy: A Prospective Cohort Study
Source: Nutrients. 2026 Nov 6;18(21):3684. doi: 10.3390/nu18213684 (PMC13765679; doi:10.3390/nu18213684)
Supplement: Supplementary file 1 [file nutrients-18-03684-s001.zip › Supplementary File S3 Family Satisfaction Assessment .pdf]

Supplementary File S3

Researcher-Developed Family Satisfaction Assessment

Metabolic, Gastric, and Psychosocial Correlates of Carbohydrate-Containing Clear-Fluid Intake in Children Undergoing Adenotonsillectomy: A Prospective Cohort Study

Administration: Before the assessment, parents were informed about both routine preoperative fasting approaches (overnight fasting and carbohydrate-containing clear-fluid intake 2 hours before surgery) and the clinical rationale for each. Parents then evaluated the approach actually applied to their child.

A. Core family satisfaction assessment (all parents)

Applied preoperative fasting approach	Overnight fasting [ ]<br>Clear fluid 2 h before surgery [ ]
Are you satisfied with the preoperative fasting/fluid approach applied to your child?	Yes [ ] No [ ]
Overall satisfaction score for the applied approach	____ / 10
Rating anchors	1 = not satisfied at all<br>10 = very satisfied

B. Additional question for the overnight fasting group

If carbohydrate-containing clear fluid 2 hours before surgery had been available as the alternative approach, would you have preferred it?	Yes [ ] No [ ]

C. Structured rating anchors for the clear-fluid group

When assigning the single overall 1–10 satisfaction score, parents were asked to consider the 10 predefined aspects below. The domains served as anchors for the overall rating; parents were asked to reduce the score from 10 when one or more aspects were viewed unfavorably. Individual domain scores were not retained or analyzed as separate quantitative outcomes.

No.	Aspect considered when assigning the overall satisfaction score
1	Ease of implementing the preoperative fasting/fluid practice
2	Managing the prescribed fluid volume (mL), including situations in which the child wanted to drink less or more than the planned amount
3	Ease of use of the fruit-juice package format
4	Whether the child liked the taste of the fruit juice
5	Family's opinion regarding the hospital's child-friendly policy
6	Family's opinion regarding shortening the preoperative fasting duration
7	Whether the child's feeling of thirst was relieved
8	Whether restlessness caused by fasting was relieved
9	Appropriateness of the timing of the child's first oral fluid intake after awakening
10	Adequacy and clarity of the instructions provided to the family

Note: This was a researcher-developed, study-specific assessment and was not formally validated. Accordingly, the family satisfaction findings were interpreted as exploratory.
